# Preliminary investigation of the effect of ferulic acid on miRNAs and LncRNAs in Mongolian horse skeletal muscle satellite cells

**DOI:** 10.3389/fgene.2025.1630614

**Published:** 2025-07-18

**Authors:** Wendian Gong, Wenqi Ding, Tugeqin Bou, Lin Shi, Yanan Lin, Xiaoyuan Shi, Zheng Li, Huize Wu, Manglai Dugarjaviin, Dongyi Bai

**Affiliations:** Key Laboratory of Equus Germplasm Innovation (Co-Construction by Ministry and Province), Ministry of Agriculture and Rural Affairs, Inner Mongolia Key Laboratory of Equine Science Research and Technology Innovation, Equus Research Center, College of Animal Science, Inner Mongolia Agricultural University, Hohhot, China

**Keywords:** ferulic acid, MuSCs, miRNA, lncRNA, muscle fiber

## Abstract

**Introduction:**

Ferulic acid (FA), a natural antioxidant, has attracted considerable attention for its regulatory potential in skeletal muscle development, energy metabolism, and muscle fiber type transformation.

**Methods:**

This study established a research system based on Mongolian horse skeletal muscle satellite cells to elucidate the molecular basis by which ferulic acid regulates muscle fiber type transformation through a non-coding RNA interaction network.

**Results:**

A total of 18 differentially expressed miRNAs (DEMIRs) and 128 differentially expressed lncRNAs (DELs) were identified through transcriptome sequencing of the ferulic acid-treated (FA) group and the control group (NC). The target genes of non-coding RNAs are enriched in processes such as positive regulation of TOR signaling, cell migration, and positive regulation of vascular endothelial cell proliferation, where they play important roles in cell growth and proliferation. Dual luciferase reporter assays confirmed that LncRNA *MSTRG.7632.14* competitively binds to *eca-miR-6529*, thereby relieving its inhibitory effect on *PDK1* and forming a biologically functional regulatory axis.

**Conclusion:**

This study reveals that ferulic acid can regulate muscle fiber proliferation and type transformation through the interaction network of non-coding RNAs and target genes, providing potential targets for optimizing the athletic performance and muscle function of equids.

## Introduction

Skeletal muscle is the primary locomotor tissue in animals, playing a key role in physiological functions such as movement, respiration, and temperature regulation (Frontera and Ochala, 2015). It exhibits strong adaptability, and its regenerative capacity relies on myosatellite cells (Guilhot et al., 2024). Myosatellite cells, as resident adult stem cells in skeletal muscle, are located between the basal lamina and the plasma membrane of muscle fibers. During muscle fiber injury and regeneration, this niche undergoes dynamic remodeling (Bentzinger et al., 2013). The formation and repair of skeletal muscle are mediated by the activation of skeletal muscle satellite cells, which proliferate, differentiate into myoblasts, and subsequently fuse with each other to form new myofibers (Zeng et al., 2022). Skeletal muscle fibers exist in a variety of forms and can be broadly divided into slow muscle fibers (type I) and fast muscle fibers (type II), with different fiber types differing in function, biochemical characteristics, and morphology (Xue et al., 2021). Type I fibers have slower contractility, higher mitochondrial content, and mainly undergo oxidative metabolism; type II fibers have faster contractility and higher glycolysis (Lee et al., 2010).

As more and more countries around the globe have begun to implement bans on antibiotic use, research on natural extracts as additives has received increasing attention. For example, natural antioxidants such as Naringin (Li et al., 2021), Grape seed proanthocyanidin extract (Xu et al., 2020), Dihydromyricetin (Guo et al., 2022) have been shown to cause a shift in the type of skeletal muscle fibers in animals. Ferulic acid, a natural antioxidant present in many fruits and vegetables, has been widely used in the food, pharmaceutical and cosmetic industries (Zduńska et al., 2018). Studies have shown that ferulic acid promotes muscle glucose uptake and regulates dysregulated redox homeostasis and metabolic pathways in iron-induced oxidative damage to the pancreas (Salau et al., 2022). In addition, ferulic acid induces the transformation of the type of skeletal muscle fibers, a phenomenon that has been observed in zebrafish (Wen and Ushio, 2017), pig (Valenzuela-Grijalva et al., 2021), and sheep (Peña-Torres et al., 2022). Previous studies have found that ferulic acid can promote the transformation of fast-twitch muscle fibers in Mongolian horses; however, its role in the regulatory mechanisms of skeletal muscle remains at an early exploratory stage, with no systematic research conducted on its regulation via non-coding RNAs.

Recent studies have shown that non-coding RNAs play key roles in regulating the respective physiological processes of organisms, especially in myofiber regulation and transformation (Panni et al., 2020). Among them, microRNAs (miRNAs) are a class of non-coding RNAs, approximately 22 nucleotides in length, that regulate gene expression at the post-transcriptional level by either inhibiting messenger RNA (mRNA) translation or promoting its degradation (Fochi et al., 2020). For example, miR-133a1, which is enriched in fast muscle, was shown to be a direct target gene of thyroid hormone in muscle, regulating slow to fast muscle fiber type conversion by targeting TEA structural domain family member 1 (*TEAD1*), where *TEAD1* is a key regulator of slow muscle gene expression (Zhang et al., 2014). Long non-coding RNAs (lncRNAs), defined as RNA transcripts exceeding 200 nucleotides in length, regulate gene expression through multiple pathways (Deniz and Erman, 2017). They can overlap with promoter regions to directly affect transcription, or influence gene expression indirectly by altering mRNA stability (Hombach and Kretz, 2016; Latos et al., 2012). Moreover, lncRNAs can function as molecular “sponges” that bind to miRNAs, competing for their interaction sites on target mRNAs, thereby diminishing miRNA-mediated gene regulation (Cesana et al., 2011; Kallen et al., 2013). Both lncRNAs and miRNAs play vital and coordinated roles in skeletal muscle contraction and development, contributing significantly to the regulation of muscle function (Archacka et al., 2021; Horak et al., 2016). Previous studies have shown that *LncRNA-FKBP1C* could promote myoblast differentiation by affecting the stability of *MYH1B*, which in turn was involved in skeletal muscle fiber type transformation (Yu et al., 2021). These non-coding RNAs often regulate skeletal muscle fiber type transformation by forming regulatory networks among lncRNAs, miRNAs, and mRNAs through the competing endogenous RNA (ceRNA) mechanism.

This study explores the regulatory mechanisms of ferulic acid on non-coding RNAs in skeletal muscle satellite cells, systematically analyzing the changes in non-coding RNA expression, regulatory networks, and functions in Mongolian horse skeletal muscle satellite cells after ferulic acid treatment, thereby providing a theoretical basis for molecular breeding of Mongolian horses.

## Materials and methods

### Culture and identification of Mongolian horse MuSCs

Skeletal muscle tissue samples were collected from three 2-year-old Mongolian horses of the same age and raised under identical conditions, and were washed with antibiotic-containing saline. The skeletal muscle cells were cultured in a medium consisting of 10% fetal bovine serum (FBS, Gibco, United Kingdom) and 90% high-glucose DMEM (Gibco, United Kingdom) in an incubator set at 37°C and 5% CO_2_. Muscle stem cells (MuSCs) were purified using the differential adhesion method. Primary cells obtained after enzymatic digestion were seeded into culture dishes and incubated for 1 h. During this period, fibroblasts and other contaminating cells adhered rapidly to the surface. The non-adherent cell suspension was then carefully transferred to a new culture dish and incubated for an additional 6 h. MuSCs selectively adhered during this period, removing the remaining contaminating cells and resulting in a highly purified MuSC population. Preliminary experiments showed that 500 ng/mL had the most significant effect. After three rounds of passage and purification, when the cells adhered and proliferated to about 30% confluence, ferulic acid was added at a concentration of 500 ng/mL, while PBS was used as the negative control. When cell proliferation reached approximately 50%, the medium was replaced with a differentiation medium containing 5% fetal bovine serum to induce differentiation. Ferulic acid was continuously added to the culture medium during differentiation. Cells were harvested when differentiation reached approximately 80%.

### Flow cytometry

The cultured skeletal muscle satellite cells were washed with PBS (5 × 10^5^ cells), then fixed overnight with 70% cold ethanol at 4°C. Samples were first treated with RNase A to eliminate RNA interference, followed by the addition of 0.5 mL propidium iodide (PI, Beyotime) and incubated for 30 min. Finally, the stained samples were analyzed using a flow cytometer (BD Accuri C6; Biosciences) at an excitation wavelength of 488 nm. Flow cytometry was used to assess cell proliferation under different treatments in the control group and the ferulic acid group. The results were visualized and analyzed using ModFit LT software (BD Biosciences).

### Library construction

Next, RNA samples were collected from three groups of independent cells. RNA sequencing (RNA-seq) libraries were constructed using the NEBNext^®^ Ultra™ Directional RNA Library Prep Kit, while small RNA sequencing (sRNA-seq) libraries were prepared with the NEBNext^®^ Multiplex Small RNA Library Prep Kit. After library preparation, purification was carried out using the AMPure XP system (Beckman Coulter, Beverly, United States). The quality of the cDNA libraries was evaluated using the Agilent Bioanalyzer 2100 system. Finally, RNA-seq libraries were sequenced on the Illumina HiSeq 2500 platform, generating 150 bp paired-end sequencing reads. Small RNA samples were sequenced on the Illumina HiSeq 2500 platform, generating 50 bp single-end reads.

### miRNA analysis and LncRNA analysis

For small RNA, Cutadapt (version 3.2) (Martin, 2011) was used to remove sequencing adapters and low-quality reads from the raw data, and FastQC (version 0.11.5) (Wingett and Andrews, 2018) was employed to assess the quality of the sequence data. Get clean sequence using Bowtie2 (version 1.1.0) (Langmead and Salzberg, 2012) and GtRNAdb (http://gtrnadb.ucsc.edu/), Rfam (https://rfam.org/), the RepBase (http://www.girinst.org/repbase/) and SILVA (https://www.arb-silva) and other databases were compared to remove unmatched sequences, repeats, tRNA, rRNA and other small Rnas. Next, the filtered sample data is compared with the reference genome using Mirdeep (version 0.0.8) (Friedländer et al., 2012), and new miRNAs are predicted based on the comparison results. Then DESeq2 (version 1.44.0) (Love et al., 2014) in R language was used for differential expression analysis of the obtained miRNA data. Finally, miRNA target gene prediction software MiRanda (Enright et al., 2003) and TargetScan (Agarwal et al., 2015) were used to predict the target by searching for the presence of conserved sites matching each miRNA seed region.

To obtain high-quality reads, the raw RNA-seq data were processed using fastp (version 0.20.0) (Chen et al., 2018) to remove adapter sequences, discard reads containing ≥10% unidentified nucleotides (N), and filter out low-quality reads with quality scores ≤Q20. The raw data have been uploaded to the Sequence Read Archive (SRA) database of the National Center for Biotechnology Information (NCBI). Clean data were aligned to the horse reference genome (https://www.ncbi.nlm.nih.gov/datasets/genome/GCF_002863925.1/) using Hisat2 (version 2.0.4) (Kim et al., 2015), and the alignment results were assembled into transcripts using StringTie (version 2.0) (Kovaka et al., 2019). To identify long non-coding RNAs, three software tools were used to screen out transcripts with protein-coding potential: CPAT (Wang et al., 2013), CNCI (Sun et al., 2013), and CPC2 (Kang et al., 2017). Subsequently, the DESeq2 software was used to conduct differential expression analysis of those transcripts that were jointly identified as non-coding by the three tools to determine the differential expression patterns of lncRNA. The genes with |log2(Fold Change)| > 1 and an adjusted false-discovery rate (FDR) < 0.05 were assigned as differentially expressed genes. Potential cis-target mRNAs were screened based on genomic location, and we searched 10 kb mRNA upstream and 10 KB mRNA downstream of different lncRNA. In this study, the Mongolian horses supplemented with ferulic acid were used as the experimental group to study the significant changes in the expression level of non-coding RNA. The prediction of trans-target mRNA was based on the Pearson correlation coefficient (r ≥ 0.95) between lncRNAs and mRNA, indicating a strong co-expression relationship.

### GO and KEGG enrichment analysis

To evaluate the biological functions and potential mechanisms of non-coding RNA target genes, Gene Ontology (GO) and Kyoto Encyclopedia of Genes and Genomes (KEGG) enrichment analyses were performed using the DAVID online tool (https://david.ncifcrf.gov/). A significance threshold of p < 0.05 was applied to identify significantly enriched functional categories and signaling pathways, thereby elucidating their roles in the biological functions of non-coding RNAs and their target genes. To further explore the changes of target genes at the overall transcriptional level, Gene Set Enrichment Analysis (GSEA) was also performed. Due to the limited annotation resources for horses, gene sets from the human Molecular Signatures Database (version GRCh3.8) were used as references.

### Interaction network between non-coding RNAs and mRNAs

To further investigate the functional roles of lncRNAs and miRNAs in the regulation of muscle satellite cells (MuSCs) in Mongolian horses, the predicted miRNA target genes and lncRNA trans-target genes were integrated and visualized using Cytoscape (version 3.7.0) for comprehensive interaction network analysis.

### Construction and identification of double luciferase

293T cells and target plasmids (*PDK1* and *MSTRG.7632.14*) divided into 96-well plates for transfection were prepared in advance, and transfection was initiated when the cell density reached 50%–70%. Dual Luciferase Reporter Assay Kit (dual-luciferase Reporter assay kit) was used for dual luciferase assay. The 20 μL lysate was added into 100 μL Luciferase Reaction Reagent which was equilibrated at room temperature, and the fLuc signal was detected in the enzymoleter after mixing. Then 100 μL of Luciferase Reaction Reagent II was added and equilibrated to room temperature, and the rLuc signal was detected in the enzymic marker after mixing, and the obtained data were statistically analyzed (*p < 0.05, **p < 0.01, ***p < 0.001).

### Fluorescence quantitative RT-PCR validation and data analysis

To validate the accuracy of the sequencing results, quantitative real-time PCR (qRT-PCR) was performed for miRNAs and lncRNAs. qRT-PCR primers were designed by Sangon Biotech Co., Ltd. (Shanghai, China) (Supplementary Table S1). For lncRNAs, total RNA was reverse-transcribed into cDNA using the HiScript^®^ II qRT SuperMix for qPCR kit (Vazyme, Nanjing, China) following the manufacturer’s instructions. qRT-PCR was conducted using the CFX96 Real-Time PCR Detection System (Bio-Rad, United States) and SYBR^®^ Premix Ex Taq™ II (TaKaRa, Japan). Due to the short sequence length of miRNAs, cDNA was synthesized from total RNA using the stem-loop miRNA first-strand cDNA synthesis kit (Vazyme, Nanjing, China), followed by real-time qPCR using the CFX96 system. The reactions were performed using specific primers and a universal miRNA SYBR qPCR master mix. The 2^(-ΔΔCt) method was used to analyze qPCR results and calculate Ct value changes among muscle samples from different horse breeds, with FDR <0.05 considered statistically significant. To ensure data reliability and accuracy, each sample was tested in triplicate.

## Results

### Ferulic acid promotes the proliferation of Mongolian horse muscle satellite cells

**FIGURE 1:**
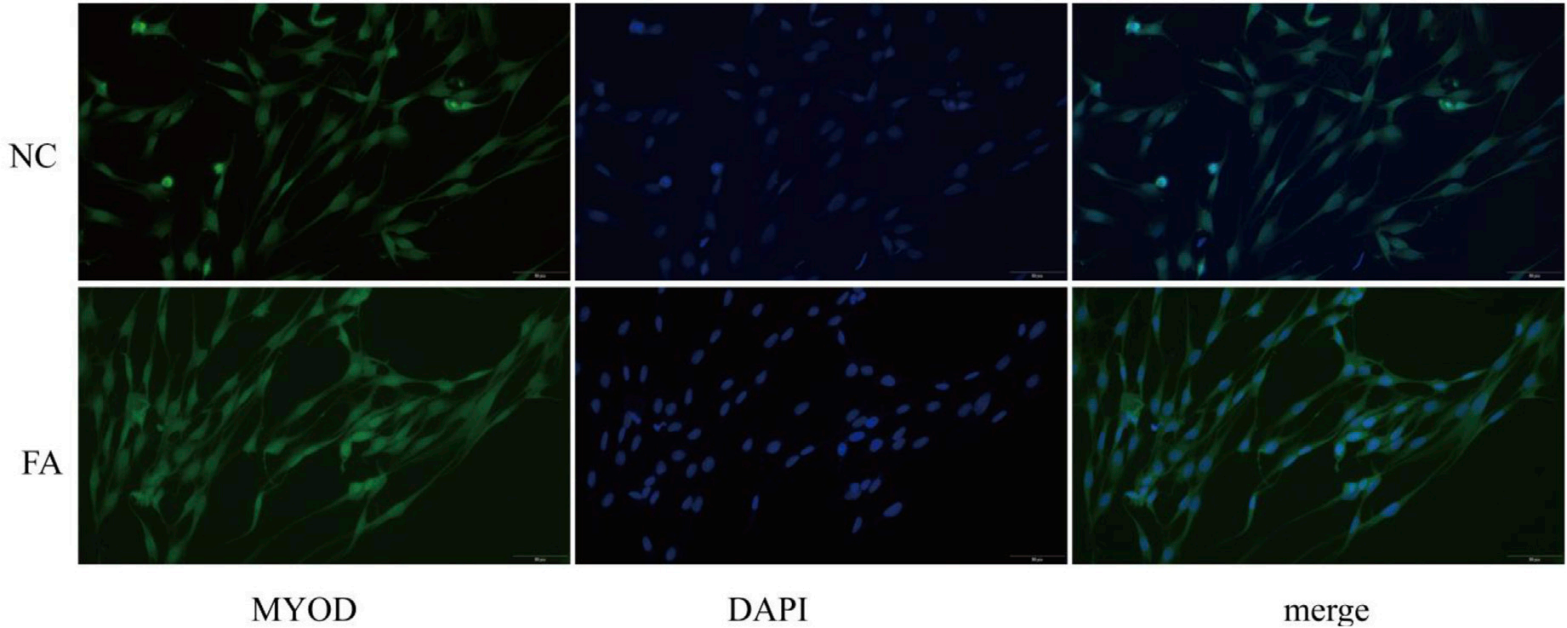
Immunofluorescence staining shows the expression of MYOD in muscle satellite cells between the ferulic acid (FA)-treated group and the negative control (NC) group.

**FIGURE 2:**
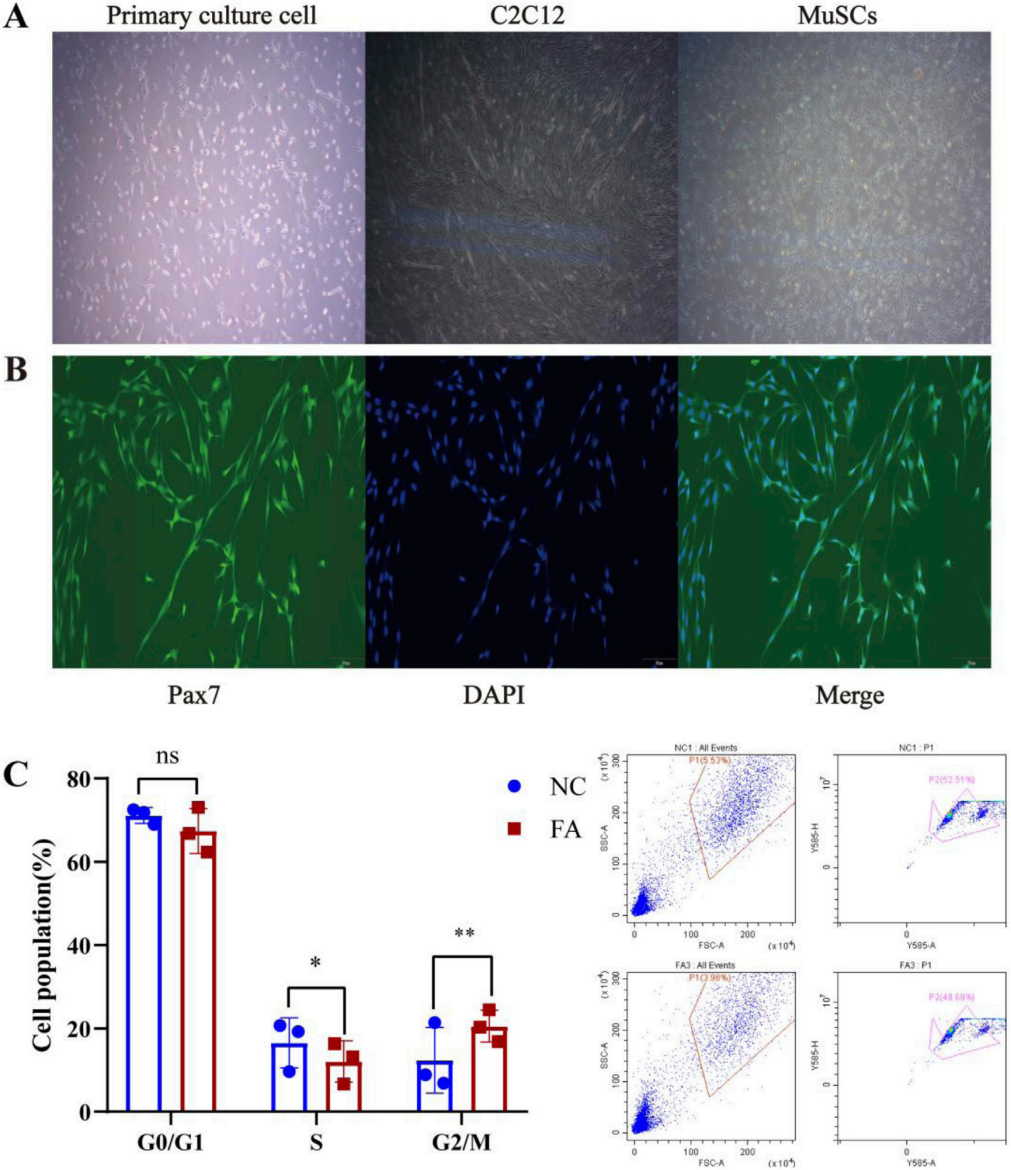
Ferulic acid enhances the proliferation of MuSCs. **(A)** Morphological changes of Mongolian horse skeletal muscle satellite cells (MuSCs) during proliferation and differentiation. **(B)** Immunofluorescence staining of the MuSC-specific marker *Pax7*. **(C)** Cell cycle distribution.

After 2 days of differentiation induction, the expression of the myogenic marker protein MYOD was assessed in both the ferulic acid-treated and control groups. Immunofluorescence analysis revealed that cells in the ferulic acid group exhibited stronger fluorescence intensity and greater fusion efficiency compared to the control. These results suggest that ferulic acid promotes the differentiation of Mongolian horse skeletal muscle satellite cells (Figure 1). The proliferating Mongolian horse skeletal muscle satellite cells (MuSCs) we cultured appeared oval in shape and were loosely arranged without an obvious orientation. As differentiation progressed, the cells gradually aggregated and aligned in a specific direction, exhibiting a typical parallel arrangement along the long axis and initiating myotube formation (Figure 2A). Immunofluorescence staining for the MuSC-specific marker *Pax7* showed a positivity rate exceeding 95%, further confirming that the isolated and cultured cells were high-purity Mongolian horse MuSCs. These results indicate that a stable and reliable *in vitro* culture system for Mongolian horse MuSCs was successfully established in this study (Figure 2B).

To investigate the effect of ferulic acid on the proliferation of skeletal muscle satellite cells (MuSCs) in Mongolian horses, our preliminary studies indicated that the proliferative effect of ferulic acid on MuSCs peaked on day 4. Therefore, cells were cultured for 4 days, and cell cycle distribution was analyzed using flow cytometry. The results showed that ferulic acid did not significantly alter the proportion of cells in the G0/G1 phase, while a marked increase was observed in the progression through the G2/M phase. This suggests that ferulic acid may enhance DNA replication by activating the cell cycle process at the G2/M checkpoint, thereby promoting the proliferative capacity of Mongolian horse MuSCs (Figure 2C).

### Identification of differential miRNAs in Mongolian horse myosatellite cells by ferulic acid

**FIGURE 3:**
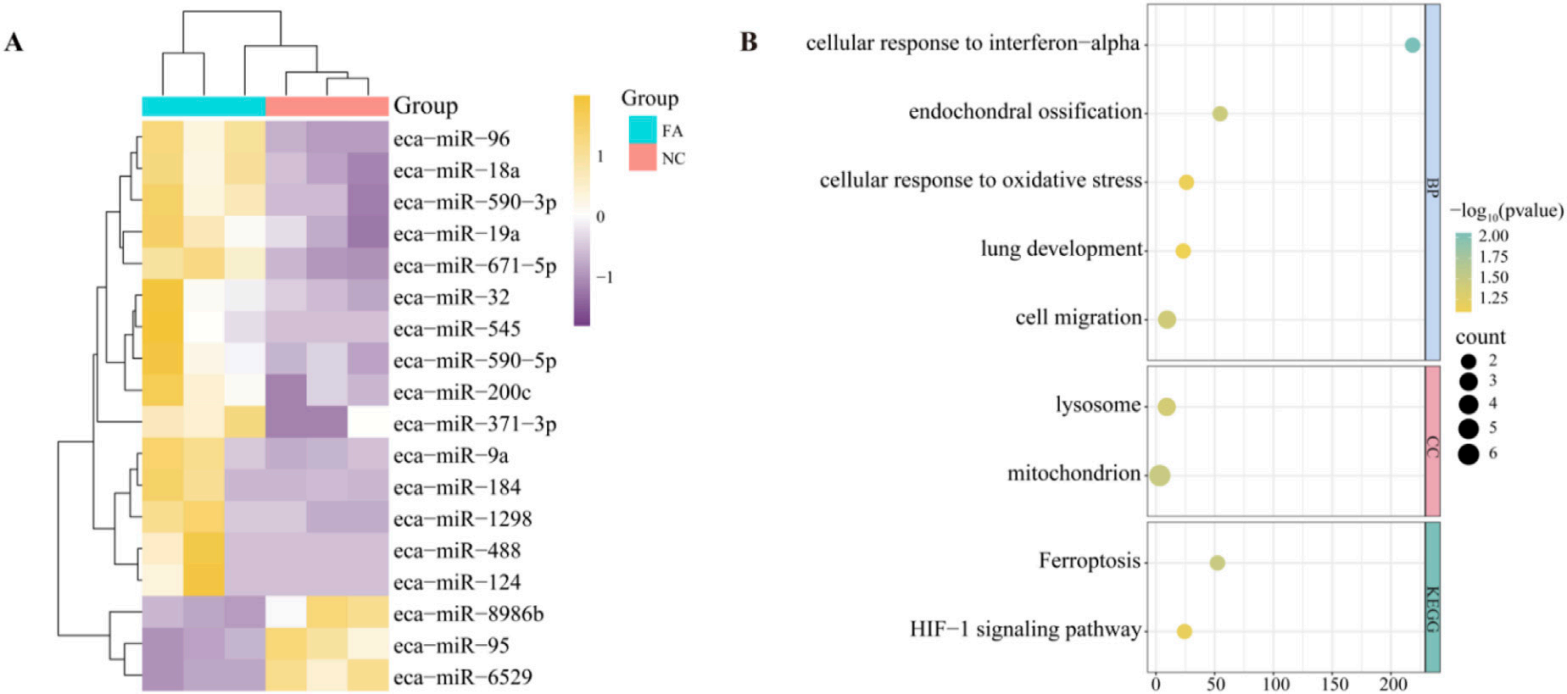
Differentially expressed miRNAs and pathway analysis. **(A)** Heatmap of differentially expressed miRNAs (DEMIRs) between the ferulic acid-treated group and the control group. **(B)** Functional enrichment analysis of target genes of DEMIRs.

In order to understand the regulation of miRNAs by ferulic acid in Mongolian horse MuScs, whole transcriptome sequencing was performed in Mongolian horse MuSCs after the addition of ferulic acid (FA) and control group (NC), and a total of 18 differential miRNAs(DEMIRs) were identified, of which 15 upregulated and 3 downregulated DEMIRs were identified (Figure 3A). Using predictive software, 15 upregulated DEMIRs were found to target 28 downregulated mRNAs, while 3 downregulated DEMIRs targeted 13 upregulated genes. To further investigate the potential biological functions of these DEMIRs, functional enrichment analysis was performed on their target genes (Figure 3B). Only two signaling pathways were enriched in the KEGG analysis, including the HIF-1 signaling pathway (ecb04066) and ferroptosis pathway (ecb04216), both of which are closely associated with muscle fiber type transformation and muscle development. GO enrichment analysis indicated that the most enriched term was cell migration (GO:0016477), which may be consistent with the physiological behavior of muscle satellite cells. Mitochondrion (GO:0005739) plays an important role in cellular energy metabolism, and cellular response to oxidative stress (GO:0034599) may play a significant role in the proliferation and differentiation of muscle cells. The enrichment of *PDK1* in the mitochondrion and HIF-1 signaling pathway may suggest that *PDK1* plays an important regulatory role in the metabolic adaptation, proliferation, and differentiation of muscle cells.

### The effect of ferulic acid on lncRNAs in Mongolian horse muscle satellite cells

**FIGURE 4:**
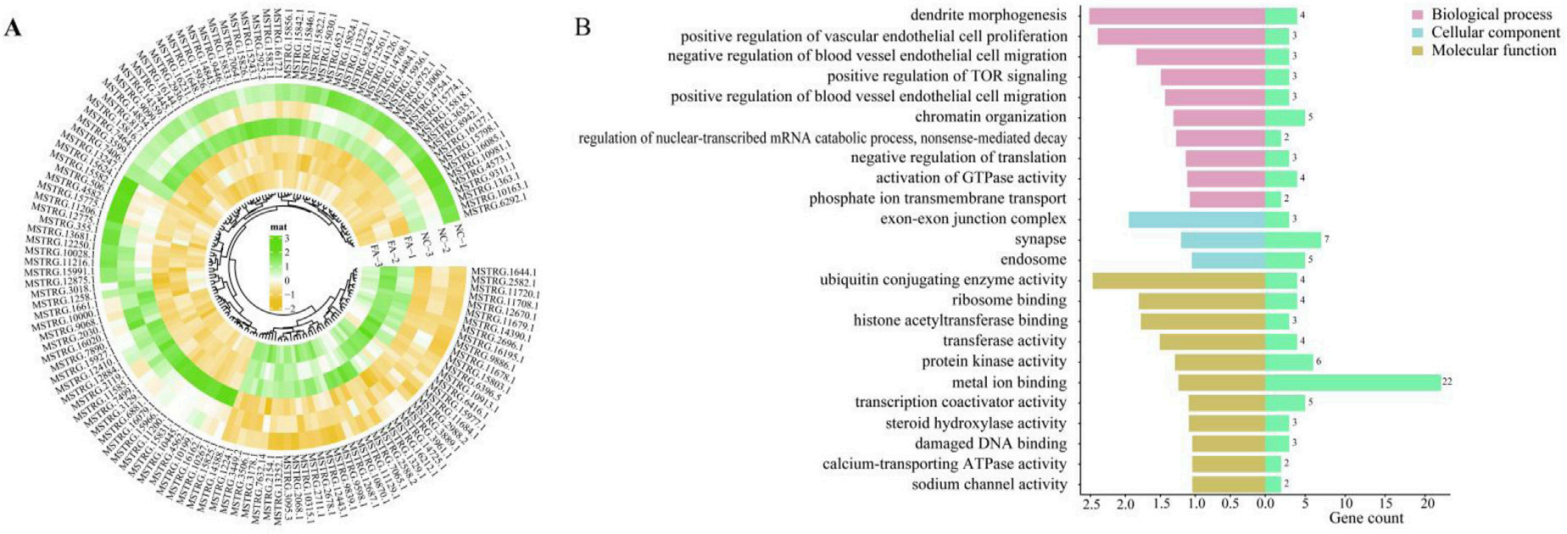
Differentially expressed lncRNAs and pathway analysis. **(A)** Heatmap of differentially expressed lncRNAs (DELs) between the ferulic acid-treated group and the control group. **(B)** Functional enrichment analysis of target genes of DELs.

A total of 228 differentially expressed lncRNAs (DELs) were identified between the FA group and NC group, including 154 downregulated and 74 upregulated lncRNAs (Figure 4A). By analyzing the potential cis-target genes within a 10 kb upstream and downstream region of these DELs, Gene Ontology (GO) enrichment revealed significant associations with pathways such as positive regulation of TOR signaling (GO:0032008), positive regulation of blood vessel endothelial cell migration (GO:0043536), and positive regulation of vascular endothelial cell proliferation (GO:1905564), These pathways work together to promote the proliferation, differentiation, and repair and regeneration of muscle tissue (Figure 4B).

### lncRNA–miRNA–mRNA interaction network

**FIGURE 5:**
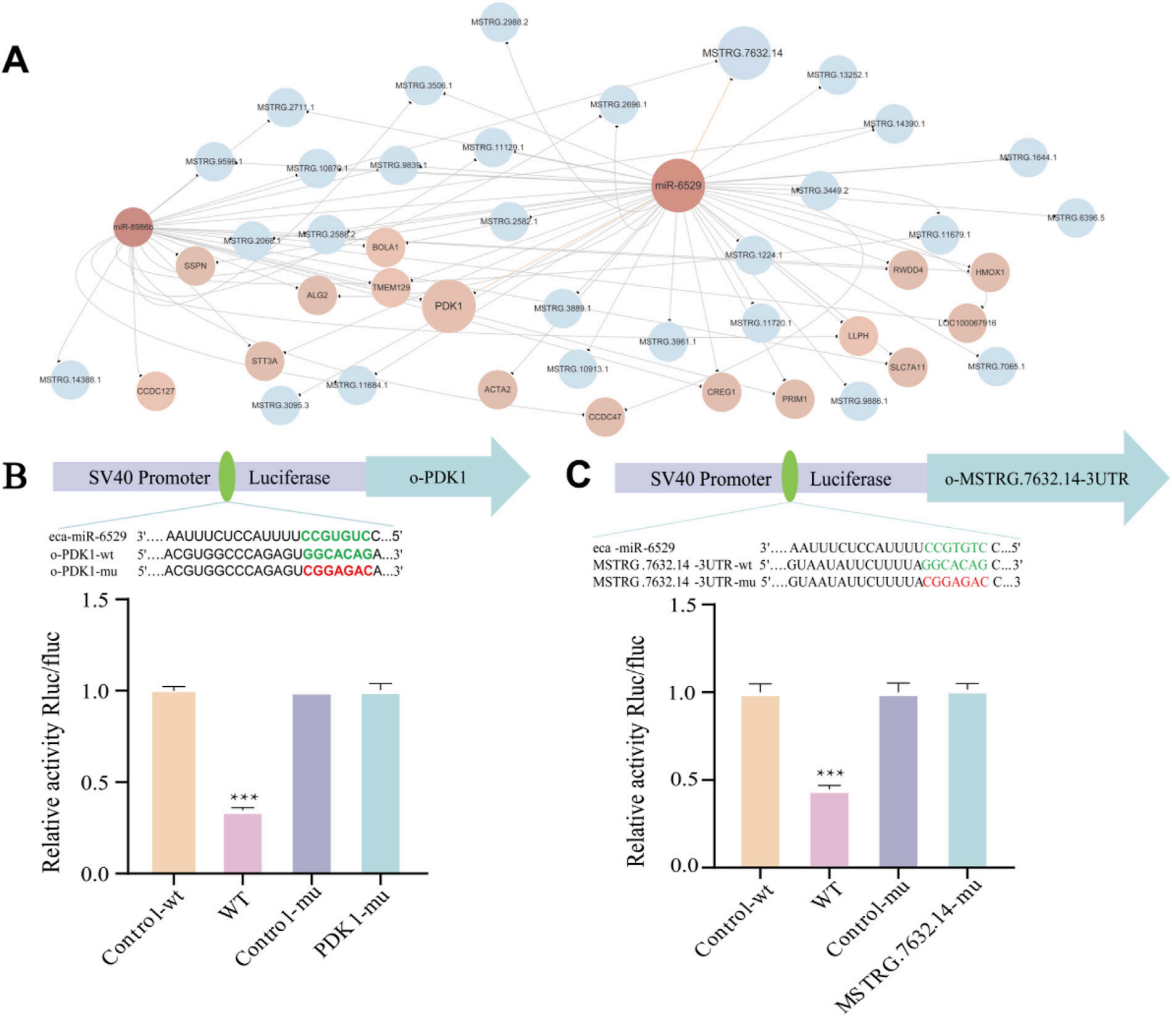
lncRNA–miRNA–mRNA interaction network and validation of key ceRNA relationships. **(A)** lncRNA–miRNA–mRNA interaction network, Blue represents lncRNA, pink represents miRNA, and light pink represents mRNA. **(B)** Predicted binding sites between *miR-6529* and *PDK1* and validation by dual-luciferase reporter assay. **(C)** Predicted binding sites between *miR-6529* and *MSTRG.7632.14* and validation by dual-luciferase reporter assay. *p < 0.05, **p < 0.01, ***p < 0.001.

To further investigate the competing endogenous RNA (ceRNA) regulatory mechanisms involved in muscle satellite cells (MuSCs) of Mongolian horses, we constructed a lncRNA–miRNA–mRNA interaction network. The analysis results showed that a total of 15 differentially expressed lncRNAs (DELs) were predicted to target *miR-8986b*, while 27 DELs were predicted to target *miR-6529*. Among them, 14 DELs were found to commonly target both miR-9886b and *miR-6529* (Figure 5A). Dual-luciferase reporter assays confirmed that *miR-6529* significantly suppressed luciferase activity driven by the wild-type 3′UTR of *MSTRG.7632.14* (p < 0.001***), while no significant suppression was observed in the mutant 3′UTR construct (p < 0.05), thereby validating the direct binding interaction between *MSTRG.7632.14* and *miR-6529* (Figure 5C).

In addition, miRNA-mRNA target prediction indicates that both *miR-8986b* and *miR-6529* target 15 genes, with 14 common mRNA targets shared between them, and both target pyruvate dehydrogenase kinase 1 (*PDK1*). This was further validated by dual-luciferase reporter assays, which demonstrated that *miR-6529* significantly repressed the luciferase activity of the wild-type *PDK1* 3′UTR construct (p < 0.001***), while the mutant version showed no significant response to *miR-6529* (p < 0.05), confirming successful mutation (Figure 5B).

Collectively, these findings suggest that FA may regulate *PDK1* expression through competitive binding between *MSTRG.7632.14* and *miR-6529*, thereby influencing the proliferation, differentiation, and fiber-type transition of muscle satellite cells. This regulatory mechanism provides important molecular insights into muscle development and adaptive evolution in Mongolian horses.

### Quantitative real-time PCR

**FIGURE 6:**
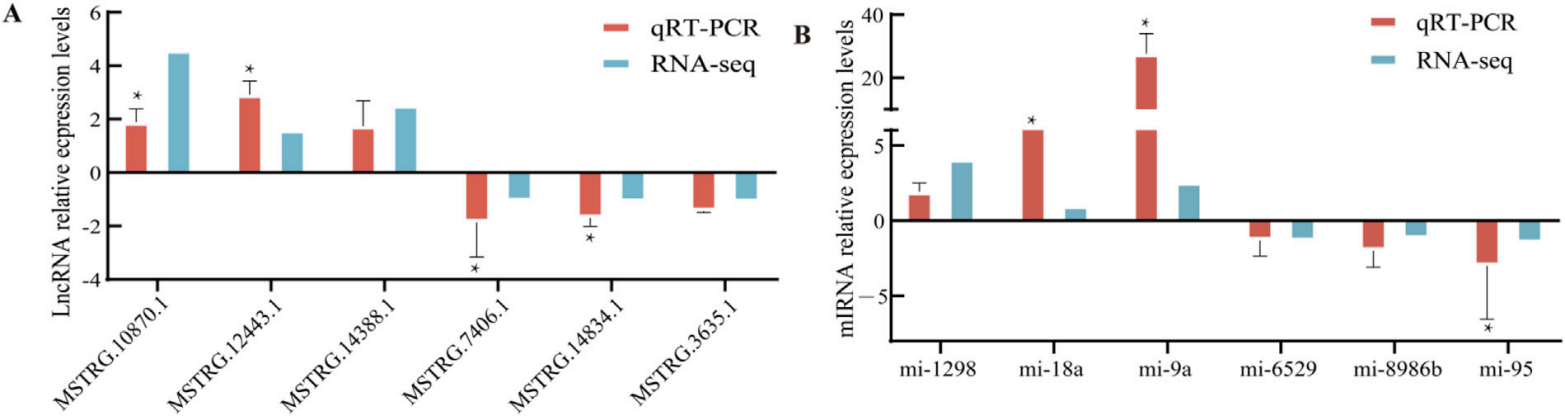
qRT-PCR validation. **(A)** Six differentially expressed lncRNAs. **(B)** Six differentially expressed miRNAs.

To validate the expression patterns of DEMIRs and DELs, a total of 12 genes were selected for verification. These included six lncRNAs (*MSTRG.10870.1*, *MSTRG.12443.1*, *MSTRG.1438.81*, *MSTRG.7406.1*, *MSTRG.14834.1*, *MSTRG.3635.1*) and six miRNAs (*miRNA-1298*, *miRNA-18a*, *miRNA-9a*, *miRNA-6529*, *miRNA-8986b*, *miRNA-95*). The expression levels of these genes were validated using qRT-PCR, and the observed expression patterns were consistent with the results of RNA-seq and sRNA-seq analyses. Statistical significance was determined based on FDR <0.05 (Figure 6). GAPDH was used as the housekeeping gene, to ensure data reliability and accuracy, each sample was tested in triplicate. Primer information can be found in Supplementary File S1.

## Discussion

Skeletal muscle remodeling is a structural and functional change that occurs during the growth and development of the organism, and myosatellites, as adult stem cells of skeletal muscle, have a regenerative function able to rebuild myogenic precursors of damaged muscle tissue (Chang and Rudnicki, 2014). Myofibers, as the main component of skeletal muscle, are involved in energy metabolism, growth and development, and adaptive transformation of skeletal muscle fiber types by interacting with MuSCs (Blaauw et al., 2013). Ferulic acid, a natural phenolic compound, has been widely used as an anti-inflammatory and antioxidant in skeletal muscle (Cavalcanti et al., 2021). In recent years, FA has now become a very popular additive in livestock farming due to its unique biological properties. Therefore, in the present study, we chose to add FA to Mongolian horse MuSCs to investigate its effect on the proliferation of equine MuSCs and to further reveal the potential role of non-coding RNAs in the regulation of skeletal muscle growth through whole transcriptome analysis.

Muscle stem cells (MuSCs) are a population of cells within adult skeletal muscle tissue that possess self-renewal and multipotent differentiation potential. They serve as a major source for new myofiber formation and are indispensable regulatory factors in the process of skeletal muscle regeneration. Under homeostatic conditions, MuSCs remain in a quiescent state, which is maintained through complex signaling interactions among various cell types. This study demonstrates that FA can effectively promote the proliferation of MuSCs, and this enhanced proliferative capacity is closely associated with improved skeletal muscle regeneration efficiency. Previous studies have shown that vitamin C can increase both the total protein level and nuclear localization of the myogenic marker gene *Pax7*. Moreover, through physical interaction with the *Pax7* protein, vitamin C enhances the expression of the *Pax7* target gene *Myf5*, thereby promoting MuSC proliferation during the regulation of post-injury skeletal muscle regeneration (Zhang et al., 2024). Upon muscle fiber injury, MuSCs are activated and enter the cell cycle, undergoing proliferation and subsequently differentiating into myotubes, which then mature into myofibers to regenerate muscle tissue (Karthikeyan and Asakura, 2024).

MiRNA plays a crucial role in cell proliferation and differentiation, exerting regulatory effects in various biological processes. Studies have shown that *miR-590-3p* (Wang et al., 2020), miR-19a (Sun et al., 2017), and *miR-1298* (Schmidt and de Wit, 2015) can promote the proliferation of cardiomyocytes and smooth muscle cells. This phenomenon is consistent with the protective effects of ferulic acid on the cardiovascular system, suggesting its potential therapeutic value in the treatment of heart diseases. Meanwhile, *miR-200c* (D’Agostino et al., 2018) and *miR-124* (Qadir et al., 2014) have been reported to inhibit cardiomyocyte differentiation, whereas *miR-95* facilitates this process (Li et al., 2017). The repair and regeneration of skeletal muscle largely depend on the dynamic balance between proliferation and differentiation of muscle satellite cells (Sousa-Victor et al., 2022). Ferulic acid may enhance the proliferation of muscle satellite cells and inhibit their premature terminal differentiation by regulating the expression of related miRNAs, thereby promoting skeletal muscle development and repair to a certain extent.

In the study of LncRNAs, the function of LncRNAs in equids could not be analyzed in depth because of the lack of studies on LncRNAs in the genus Equus. However, enrichment analysis showed that LncRNAs are associated with more pathways than miRNAs, including GTPase activity, calcium-transporting ATPase activity and ubiquitinating enzyme activity, etc. ATP and GTP are essential for muscle contraction and metabolism (Hibberd et al., 1985), and GTPase plays an important role in the development, regeneration and metabolic homeostasis of skeletal muscle (Rodríguez-Fdez and Bustelo, 2021). In addition, ferulic acid may promote myofiber growth and type conversion by regulating enzyme activities such as protein kinase and ubiquitinase.

**FIGURE 7:**
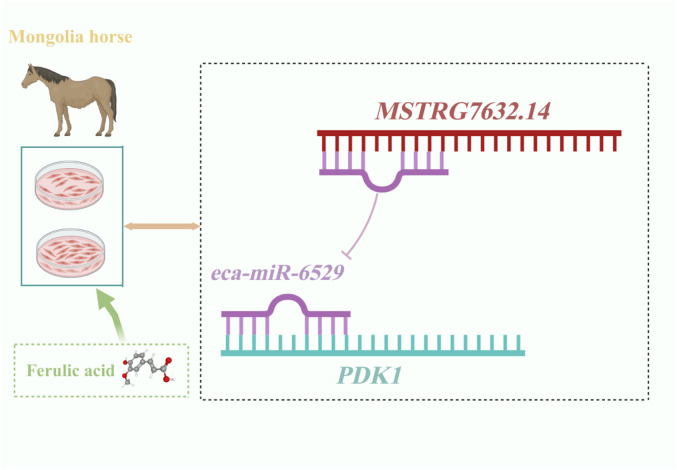
Regulation model of MuSCs specific non-coding RNA by ferulic acid in Mongolian horse.

In this study, we further analyzed the ceRNA network regulated by ferulic acid, and found that *PDK1* was the key target gene. *eca-miR-6529* and eca-*miR-8986b* could target *PDK1*, and the experimental results showed that *eca-miR-6529* had a stronger binding ability. In addition, LncRNA-*MSTRG.7632.14* has the most binding sites with *eca-miR-6529*, and the experiments verified its direct regulatory relationship, suggesting that ferulic acid can inhibit *eca-miR-6529* through upregulation of *MSTRG.7632.14*, thus promoting *PDK1* expression and affecting myofiber growth (Figure 7). Additionally, several non-coding RNAs were found to target *HMOX1*, and both *HMOX1* and *PDK1* were enriched in the HIF-1 signaling pathway. Although we have explored the functions and associated pathways of non-coding RNA target genes through GO and KEGG enrichment analyses, we also recognize that Gene Set Enrichment Analysis (GSEA) can provide additional insights based on the entire gene expression profile. The results showed that the *PDK1* gene was enriched in processes such as oxidative stress, energy metabolism, and cell proliferation, suggesting that *PDK1* may be a key candidate functional gene involved in regulating metabolism and cell survival during exercise-related stress in horses. Meanwhile, *HMOX1* was significantly enriched in multiple pathways, indicating that it may play a regulatory role in various biological processes or signaling pathways, exhibiting considerable multifunctionality. Previous studies have shown that *HMOX1* is an anti-inflammatory and antioxidant enzyme that can promote myofibroblast differentiation by inhibiting muscle injury, and its expression is reduced in muscular atrophy. *HMOX1* has been reported to have potential therapeutic value in maintaining muscle contractile function (Pietraszek-Gremplewicz et al., 2018). Based on the findings of this study, ferulic acid may promote skeletal muscle growth, development, and fiber-type transformation by simultaneously upregulating *PDK1* and *HMOX1*.

This study was mainly conducted under *in vitro* conditions using muscle satellite cells (MuSCs) from Mongolian horses. However, *in vitro* culture conditions cannot fully replicate the complex physiological environment of the organism, which presents a limitation of the study. *In vivo* experimental validation is lacking; therefore, further animal studies are necessary to confirm these findings under physiological conditions. Additionally, this study found that the *MMP14* gene can promote myogenesis and regeneration (Snyman and Niesler, 2015). Taylor et al. discovered that *MMP14* plays an important role in tendon development by releasing collagen fibers from fibripositors and promoting the formation of new fibers (Taylor et al., 2011). Multiple miRNAs, including *miRNA-18a* and *miRNA-96*, target this gene. These miRNAs also play important roles in the process of cell proliferation (Hu et al., 2014; Rahimi et al., 2022), suggesting that ferulic acid may exert its function by regulating mRNA through these miRNAs.

Although this study conducted a relatively systematic analysis, several limitations remain. First, although a ceRNA regulatory network was constructed and potential key regulatory axes were proposed, comprehensive experimental validation of the network is still lacking. The current findings are mainly based on expression correlations, sequence complementarity predictions, and dual-luciferase reporter assay predictions, and thus require further confirmation through molecular biology techniques such as RNA immunoprecipitation (RIP) or gene knockdown/overexpression. In addition, alternative splicing plays an important role in regulating gene function and muscle performance and may contribute to the adaptation of sport horses to training and environmental changes. Future studies could incorporate long-read sequencing or more advanced RNA sequencing technologies to investigate transcript isoforms, thereby providing deeper insights into the complexity of transcriptional regulation. These tools can help reveal broader biological mechanisms and potential upstream regulators and are worth incorporating in future research.

Currently, research on the biological functions of ferulic acid in the skeletal muscle of equine species remains limited. This study reveals the regulatory role of ferulic acid in skeletal muscle satellite cells (MuSCs) of Mongolian horses, demonstrating that ferulic acid participates in the regulation of muscle cell proliferation and muscle fiber type transformation through the *MSTRG.7632.14*–*eca-miR-6529*–*PDK1* ceRNA network. This finding provides new theoretical evidence for the potential application of ferulic acid in muscle repair and enhancement of athletic performance. Future studies may further explore the specific mechanisms by which ferulic acid influences skeletal muscle growth in Mongolian horses. The current study primarily focuses on the computational prediction of ceRNA interactions. To enhance the reliability of the ceRNA network, future studies will incorporate additional experimental validation, with a focus on verifying the interaction mechanisms between miRNAs and their target genes, as well as between lncRNAs and their target genes. In addition, various analytical tools will be used to further investigate the specific functions and roles of different non-coding RNAs in biological processes. This study lays a theoretical foundation for understanding the role of ferulic acid in muscle development and fiber type regulation in Mongolian horses, and it offers new insights into its potential applications in sport horse breeding and the treatment of muscle-related diseases.

## Conclusion

This study analyzed the effects of ferulic acid on skeletal muscle satellite cells from Mongolian horses and found significant changes in the expression of miRNAs and lncRNAs, specifically 18 differentially expressed miRNAs (DEMIRs) and 228 differentially expressed lncRNAs (DELs). The target genes involved in these changes are associated with several key biological pathways, including the TOR signaling pathway, cell migration, and endothelial cell proliferation, all of which are crucial for muscle cell growth and function. Additionally, dual-luciferase reporter assays confirmed that *MSTRG.7632.14* alleviated the inhibitory effect of *eca-miR-6529* on *PDK1* by competing for binding, thereby promoting muscle fiber development. These findings highlight the important role of miRNAs and lncRNAs in regulating muscle fiber proliferation and type transformation by ferulic acid, providing valuable insights into the molecular pathways that regulate muscle function and laying the foundation for the development of potential targets to improve athletic performance and muscle health in horses.

## Data Availability

The datasets presented in this study can be found in online repositories. The names of the repository/repositories and accession number(s) can be found in the article/Supplementary Material.

## Abbreviations

FA, Ferulic acid; lncRNA, Long non-coding RNA; miRNA, MicroRNA; *PDK1*, 3-Phosphoinositide-dependent protein kinase-1; TOR, Target of rapamycin.
