# Supplementary material for: Preliminary investigation of the effect of ferulic acid on miRNAs and LncRNAs in Mongolian horse skeletal muscle satellite cells
Source: Front Genet. 2025 Jul 18;16:1630614. doi: 10.3389/fgene.2025.1630614 (PMC12314428; doi:10.3389/fgene.2025.1630614)

| TERM | Genes |
| --- | --- |
| GO_HOMEOSTATIC_PROCESS | HMOX1;AXL;PRIM1;SCARA5;CCDC47;CD34 |
| GO_RESPONSE_TO_OXIDATIVE_STRESS | HMOX1;PDK1;SRXN1;SLC7A11;MMP14;TOR1A |
| GO_CELLULAR_RESPONSE_TO_STRESS | PDK1;TMEM33;HMOX1;SCARA5;TOR1A;CCDC47 |
| GO_REGULATION_OF_VESICLE_MEDIATED_TRANSPORT | HMOX1;TOR1A;CDH13;LAMP1;AXL |
| GO_SINGLE_ORGANISM_CATABOLIC_PROCESS | HMOX1;PDK1;GM2A;TOR1A;MMP14;CCDC47 |
| GO_CATABOLIC_PROCESS | CCDC47;TOR1A;MMP14;HMOX1;GM2A;PDK1 |
| GO_WOUND_HEALING | CD34;AXL;PLAT;HMOX1;SLC7A11;TOR1A |
| GO_RESPONSE_TO_WOUNDING | HMOX1;CD34;AXL;PLAT;SLC7A11;TOR1A |
| GO_RESPONSE_TO_ENDOGENOUS_STIMULUS | SLC7A11;TYMS;MMP14;CDH13;FGF18;HMOX1 |
| GO_RESPONSE_TO_ABIOTIC_STIMULUS | PDK1;HMOX1;PLAT;SCARA5;MMP14 |
| GO_REGULATION_OF_TRANSCRIPTION_FROM_RNA_POLYMERASE_II_PROMOTER | KANK2;TCEA3;SUB1;CDH13;ARID5B;HMOX1;CREG1 |
| GO_CELL_PROLIFERATION | CREG1;TYMS;PDK1;HMOX1;CD34;CDH13;MMP14 |
| GO_EPITHELIUM_DEVELOPMENT | ACTA2;TYMS;MMP14;SLC7A11;TOR1A;CD34 |
| GO_CONNECTIVE_TISSUE_DEVELOPMENT | TYMS;ACTA2;FGF18;ARID5B;CD34 |
| GO_REGULATION_OF_RESPONSE_TO_STRESS | LAMP1;SCARA5;PLAT;IFNAR1;TMEM33;CD34 |
| GO_POSITIVE_REGULATION_OF_GENE_EXPRESSION | ACTA2;SUB1;IFNAR1;CD34;CDH13;ARID5B |
| GO_REGULATION_OF_RESPONSE_TO_EXTERNAL_STIMULUS | PLAT;FGF18;KANK2;CD34;SCARA5;CDH13 |
| GO_ENZYME_LINKED_RECEPTOR_PROTEIN_SIGNALING_PATHWAY | ARID5B;FGF18;AXL;PLAT;SUB1 |
| GO_POSITIVE_REGULATION_OF_MULTICELLULAR_ORGANISMAL_PROCESS | MMP14;FGF18;AXL;HMOX1;IFNAR1;CD34 |
| GO_CELLULAR_RESPONSE_TO_ORGANIC_SUBSTANCE | IFNAR1;TOR1A;CDH13;FGF18;AXL |
| GO_POSITIVE_REGULATION_OF_TRANSPORT | AXL;CD34;TOR1A;LAMP1;IFNAR1 |
| GO_TISSUE_DEVELOPMENT | FGF18;ARID5B;MMP14;TOR1A;CD34;SLC7A11;TYMS;ACTA2 |
| GO_BLOOD_VESSEL_MORPHOGENESIS | CD34;FGF18;HMOX1;MMP14;CDH13 |
| GO_ANGIOGENESIS | FGF18;CD34;MMP14;CDH13;HMOX1 |
| GO_ANATOMICAL_STRUCTURE_FORMATION_INVOLVED_IN_MORPHOGENESIS | CDH13;MMP14;FGF18;CD34;HMOX1 |
| GO_CELL_MOTILITY | PLAT;AXL;CD34;ARID5B;SLC7A11;MMP14;CDH13;TOR1A |
| GO_CIRCULATORY_SYSTEM_DEVELOPMENT | FGF18;CD34;MMP14;CDH13;ACTA2;HMOX1 |
| GO_MOVEMENT_OF_CELL_OR_SUBCELLULAR_COMPONENT | CDH13;TOR1A;MMP14;ARID5B;PLAT;AXL;SLC7A11;CD34 |
| GO_NEGATIVE_REGULATION_OF_GENE_EXPRESSION | CD34;KANK2;ARID5B;MAF1;HMOX1 |
| GO_REGULATION_OF_MULTICELLULAR_ORGANISMAL_DEVELOPMENT | HMOX1;MMP14;AXL;FGF18;CD34 |
| GO_POSITIVE_REGULATION_OF_DEVELOPMENTAL_PROCESS | MMP14;FGF18;AXL;HMOX1;CD34 |
| GO_VASCULATURE_DEVELOPMENT | HMOX1;ACTA2;MMP14;CDH13;CD34;FGF18 |
| GO_REGULATION_OF_CELLULAR_LOCALIZATION | IFNAR1;CD34;TOR1A;LAMP1;HMOX1 |
| GO_CELL_DEVELOPMENT | TOR1A;ARID5B;FGF18;AXL;ACTA2;TYMS |
| GO_PROTEIN_LOCALIZATION | SELENBP1;RND3;LAMP1;TOR1A;TMEM33 |
| GO_LOCOMOTION | ARID5B;PLAT;AXL;MMP14;LAMP1;CDH13;TOR1A;CD34;SLC7A11 |
| GO_INTRACELLULAR_SIGNAL_TRANSDUCTION | PDK1;IFNAR1;HMOX1;FGF18;AXL;CDH13;RND3 |
| GO_NEGATIVE_REGULATION_OF_RESPONSE_TO_STIMULUS | KANK2;CD34;HMOX1;MMP14;PLAT |
| GO_POSITIVE_REGULATION_OF_BIOSYNTHETIC_PROCESS | IFNAR1;SUB1;ARID5B;CDH13;HMOX1 |
| GO_REGULATION_OF_IMMUNE_SYSTEM_PROCESS | HMOX1;AXL;LAMP1;MMP14;CD34;IFNAR1 |
| GO_NEGATIVE_REGULATION_OF_NITROGEN_COMPOUND_METABOLIC_PROCESS | MAF1;ARID5B;HMOX1;CREG1;CD34;KANK2 |
| GO_IMMUNE_SYSTEM_PROCESS | CD34;IFNAR1;SLC7A11;HMOX1;AXL |
| GO_REGULATION_OF_TRANSPORT | CD34;IFNAR1;AXL;LAMP1;TOR1A;CDH13;HMOX1 |
| GO_POSITIVE_REGULATION_OF_CELL_COMMUNICATION | TMEM33;HMOX1;CDH13;TOR1A;AXL;FGF18 |
| GO_REGULATION_OF_CELLULAR_COMPONENT_MOVEMENT | MMP14;LAMP1;CDH13;FGF18;HMOX1 |
| GO_BIOLOGICAL_ADHESION | RND3;TOR1A;CDH13;AXL;IFNAR1;SLC7A11;CD34;SSPN |
| GO_REGULATION_OF_BODY_FLUID_LEVELS | SLC7A11;SCARA5;CD34;AXL;PLAT |
| GO_POSITIVE_REGULATION_OF_RESPONSE_TO_STIMULUS | TMEM33;AXL;FGF18;LAMP1;CDH13;HMOX1 |
| GO_PROTEIN_COMPLEX_SUBUNIT_ORGANIZATION | TMEM33;SCARA5;TOR1A;MAF1;HMOX1 |
| GO_MACROMOLECULAR_COMPLEX_ASSEMBLY | TMEM33;TOR1A;SCARA5;MAF1;HMOX1 |
| GO_PROTEIN_COMPLEX_BIOGENESIS | SCARA5;TOR1A;MAF1;HMOX1;TMEM33 |
| GO_SYSTEM_PROCESS | HMOX1;ACTA2;SSPN;GM2A;CD34 |
| GO_RESPONSE_TO_LIPID | AXL;IFNAR1;MMP14;HMOX1;TYMS |
| GO_RESPONSE_TO_EXTERNAL_STIMULUS | AXL;MMP14;HMOX1;IFNAR1;ACTA2;TYMS |


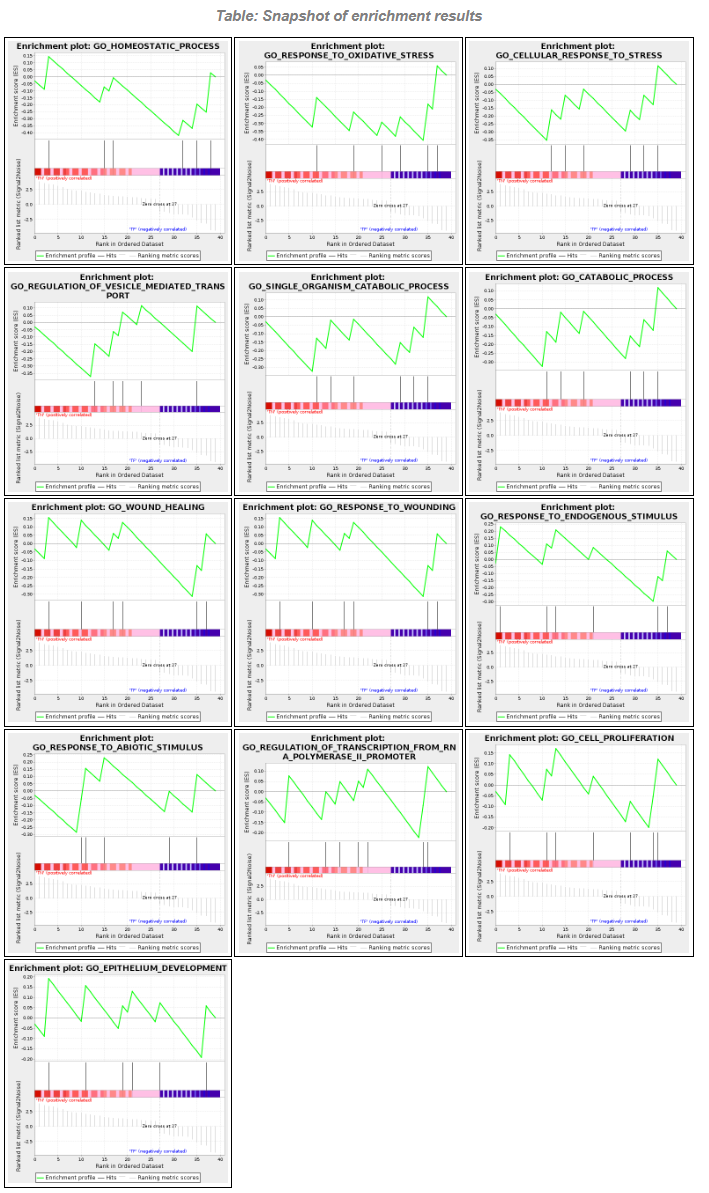

Supplement: Supplementary file 1 [file DataSheet1.zip › Supplementary Materials/Supplementary Table S4 GSEA Functional Enrichment Results.docx]
